# Sexual function in multiple sclerosis and associations with demographic, disease and lifestyle characteristics: an international cross-sectional study

**DOI:** 10.1186/s12883-016-0735-8

**Published:** 2016-11-04

**Authors:** Claudia H. Marck, Pia L. Jelinek, Tracey J. Weiland, Jane S. Hocking, Alysha M. De Livera, Keryn L. Taylor, Sandra L. Neate, Naresh G. Pereira, George A. Jelinek

**Affiliations:** 1 Neuroepidemiology Unit, Centre for Epidemiology and Biostatistics, Melbourne School of Population and Global Health, University of Melbourne, 207 Bouverie Street, Carlton, 3065 VIC Australia; 2 School of Medicine, Notre Dame University, Fremantle, WA Australia; 3 Sexual Health Unit, Melbourne School of Population and Global Health, University of Melbourne, Melbourne, VIC Australia; 4 Biostatistics Unit, Melbourne School of Population and Global Health, University of Melbourne, Melbourne, VIC Australia; 5 Emergency Department, Box Hill Hospital, Box Hill, VIC Australia

**Keywords:** Multiple sclerosis, Sexual function, Sexual dysfunction, Epidemiology, Prevalence

## Abstract

**Background:**

Sexual dysfunction (SD) is very common in people with multiple sclerosis (PwMS) and contributes a significant burden of disease, particularly for young people. SD has direct neurological contributions from depression and fatigue, which occur commonly in PwMS. Modifiable factors may represent potential targets for treatment and prevention of SD. We aimed to assess the prevalence of SD and explore associations between SD and demographic and modifiable risk factors, as well as depression and fatigue in a large cohort of PwMS.

**Methods:**

We analysed self-reported data from a large, international sample of PwMS recruited via Web 2.0 platforms, including demographic, lifestyle and disease characteristics. Specific sexual function questions included 4 items from the sexual function scale and 1 item regarding satisfaction with sexual function, part of the MS Quality of Life-54 instrument.

**Results:**

2062 PwMS from 54 countries completed questions on sexual function. 81.1 % were women, mean age was 45 years, most (62.8 %) reported having relapsing-remitting MS. The majority (54.5 %) reported one or more problems with sexual function and were classified as having SD. Lack of sexual interest (41.8 % of women), and difficulty with erection (40.7 % of men) were most common. The median total sexual function score was 75.0 out of 100, and 43.7 % were satisfied with their sexual function. Regression modeling revealed independent associations between sexual function and satisfaction and a range of demographic factors, including age, as well as depression risk, antidepressant use, and fatigue in PwMS.

**Conclusion:**

This cross-sectional study shows that SD and lack of satisfaction with sexual function are associated with depression risk and fatigue, as well as modifiable lifestyle factors diet and physical activity (after adjusting for depression and fatigue). Planned longitudinal follow-up of this sample may help clarify these associations and the underlying mechanisms. There is potential to prevent and treat SD in PwMS by addressing depression and fatigue and their determinants. Clinicians and PwMS should be aware of SD and associated factors as part of a comprehensive preventive approach to managing MS.

## Background

Multiple sclerosis (MS) is the most common disabling condition of young adults with myriad potential effects on neurological function, including sensory and autonomic function [1]. Subtle neurological disturbances can directly affect sexual function at a time of life when it assumes particular importance for many people. MS can also affect sexual function indirectly through other physical disabilities such as spasticity or bladder dysfunction, as a side effect of medications and through psychosocial mechanisms such as the presence of depression [2]. Disturbed sexual function can have detrimental effects particularly on partner relationships and mental health-related quality of life, well demonstrated in a large North American Research Committee on Multiple Sclerosis (NARCOMS) Registry sample of 6,183 PwMS [3].

Sexual dysfunction (SD) is very common in people with MS (PwMS). In reviewing seven prior studies with a total of 455 women and 326 men with MS, a 1995 review reported a prevalence of 33–75 % for females and 47–75 % for males [4]. This review has often been cited in subsequent papers as a prevalence of 40 to 80 % for females and 50 to 90 % for males [5–9]. More precise prevalence of SD is difficult to determine due to inconsistent definitions. The need for standard definitions has been recognised and specific indices proposed [10–12]. These include definitions for sexual arousal disorders in women, erectile dysfunction in men, orgasmic dysfunction and dyspareunia. There is not, however, a consistent definition in the literature for the diagnosis of SD [9].

According to the World Health Organization (WHO), SD is a syndrome that includes one or more of: lack or loss of sexual desire, sexual aversion and lack of sexual enjoyment, failure of genital response (erectile dysfunction in men and vaginal dryness or failure of lubrication in women), orgasmic dysfunction, premature ejaculation, vaginismus and dyspareunia [13].

It has been proposed that MS can affect sexual function through a variety of mechanisms, both direct and indirect [7, 14–18], however the exact aetiology is still a matter of debate [7, 9, 16, 19]. Primary SD refers to direct impairment of sexual responses or feelings by neurological damage in the central nervous system. This may lead to symptoms such as erectile dysfunction, anorgasmia and decreased sensation. Secondary SD is when physical changes indirectly affect sexual responses, unrelated to specific nerve pathways to the genitals, including fatigue, muscle tightness, spasticity or weakness and bladder or bowel dysfunction. Tertiary SD relates to psychosocial issues associated with body image, emotional challenges and cultural influences, including the commonly experienced effects of depression on sexual function and low self-esteem that indirectly affect sexual function [8, 9, 16, 19–21]. Several studies have identified demographic variables associated with sexual dysfunction, including age, number of children, education, and relationship duration, while disease characteristics such as level of disability, duration and type of MS also play a role [21–23].

The importance of SD to the quality of life of PwMS and its association with depression has been highlighted in previous research [24], but there is little knowledge about potentially modifiable lifestyle determinants of sexual function and dysfunction for PwMS. This study aimed to assess prevalence of SD and satisfaction with sexual function in an international sample of PwMS recruited online as part of the Health Outcomes and Lifestyle In a Sample of people with Multiple sclerosis (HOLISM) study. We aimed to assess the associations of disease characteristics, fatigue, depression risk and demographics with sexual function and satisfaction, and explore potential independent associations with modifiable lifestyle factors.

## Methods

### Participants and data collection

The methodology of the HOLISM study has previously been described in detail [25]. In brief, participants were recruited via online platforms, including social media, websites and forums that engaged people with MS, most of which had a health and lifestyle focus. The web-based tool, SurveyMonkey®, was used to provide respondents with a participant information sheet, an electronic consent indicator, and the survey itself. Inclusion criteria were adults of 18 years of age or more, self-reporting a formal diagnosis of MS by a medical doctor. As the survey was in English, only English speaking participants were included. Contact details were recorded for planned longitudinal follow up.

### Ethics, consent and permissions

The Health Sciences Human Ethics Sub-Committee at the University of Melbourne provided ethical approval for the study (Ethics ID: 1545102). Participants were asked to read the participant information and to consent before entering the survey.

### Data collected and tools used

The online survey used validated tools where possible, and took approximately 40 min to complete. Specific to this study were items exploring demographic variables, morbidity indicators and modifiable lifestyle factors.

### Sexual function and satisfaction

The Multiple Sclerosis Quality Of Life (MSQOL-54) sexual function scale and satisfaction item were used to assess these variables. The sexual function scale consists of 4 items regarding: lack of sexual interest, difficulty with erection (for men)/lubrication (for women), difficulty having orgasm, and ability to satisfy a sexual partner. These items had a drop-down menu with 4 options (and corresponding score): “not a problem” (100), “a little of a problem” (66.6), “somewhat of a problem” (33.3), and “very much a problem” (0). These scores are added and divided by the number of items which gives rise to the sexual function score of 0–100, if 2 or more items were completed. We categorised participants as having SD when “somewhat of a problem” or “very much a problem” was selected in response to one or more of the four items, and 2 or more out of the 4 items were completed. In addition, satisfaction was assessed with a single item: “Overall, how satisfied were you with your sexual function during the past 4 weeks?” which had a drop-down menu with 5 options which were merged in two categories: “satisfied” (comprising “very satisfied” and “somewhat satisfied”) and “not satisfied” (comprising “neither satisfied nor dissatisfied”, “somewhat dissatisfied” and “very dissatisfied”).

### Demographic factors

Demographic factors were collected using researcher-devised items and included age, gender, marital status, number of children, educational status, and occupational status.

### Morbidity indicators

Health-related Quality of Life (HRQOL) was assessed by the widely used MSQOL-54, giving rise to the mental and physical composite scores, the sexual function scale, as well as several other scales that were not included in these analyses. Level of gait disability was assessed using the Patient Determined Disease Steps (PDDS), a self-reported surrogate tool to the Expanded Disability Status Scale (EDSS), which has ordinal scores from 0 (no disability) to 8 (bed-bound). Researcher-devised items assessed self-reported doctor-diagnosed relapse rate and type of MS currently diagnosed. A list of 24 disease-modifying drugs (DMDs) was provided and participants were categorised as either currently using a DMD, or not, and in addition participants were asked if they were currently using antidepressants.

### Modifiable Lifestyle factors

Height and weight information allowed calculation of body mass index (BMI) according to the World Health Organisation (WHO) criteria (<20 = underweight, 20–25 = normal weight, 25–30 = overweight and >30 = obese). Dietary habits were assessed with the Dietary Habits Questionnaire (DHQ) on a scale of 0–100. The International Physical Activity Questionnaire (IPAQ) was included to assess level of regular physical activity (low, moderate and high). Omega-3 and vitamin D supplementation (yes/no), meditation practice (never, ≤once a week, >once a week), level of alcohol consumption (low (<15 g/week), moderate (up to 30 g/day for females; up to 45 g/day for men), or high) and current smoking status (yes/no) were assessed using researcher-devised items. All data were self-reported.

### Data analysis

Data were analysed using Stata 13 (StataCorp. 2013). Continuous data were summarised using mean (standard deviation) or median (interquartile range), and categorical data using number (percentage).

Multivariable logistic regression models were used to investigate factors associated with SD and satisfaction with sexual function. Multivariable linear regression modeling was used to investigate factors associated with sexual function score. Models were built using forward variable selection [26]. Variables explored included stable demographic factors, clinical factors and modifiable lifestyle factors: age (continuous), gender, marital status, number of children, employment status, education status, level of disability, current use of disease modifying drug, current use of antidepressants, depression screen, clinically significant fatigue, smoking status, level of alcohol use, level of physical activity, omega 3 supplementation, vitamin D supplementation, meditation frequency, body mass index, and dietary score.

Odds ratios and 95 % CIs are reported for logistic regression and coefficients and 95%CI are reported for linear regression. We used complete case analyses, that is analyses restricted to available data.

## Results

### Participation, demographics, and sexual function

Of 2469 survey respondents with confirmed MS, a total of 2062 completed sexual function items enabling derivation of a sexual function scale score and categorization of SD (yes/no). Demographic and clinical variables are described in Table 1.

**Table 1:** Demographic and clinical variables

Demographic and clinical variables		N^a^ (%)
Gender	Female	1663 (81.1)
	Male	388 (18.9)
Age groups	18–39 years	648 (32.4)
	40–49 years	665 (33.2)
	>49 years	690 (34.4)
Country of residence	United States of America	691 (33.5)
	Australia	526 (25.5)
	United Kingdom	339 (16.4)
	51 other countries	506 (24.5)
Marital status	Married, cohabitating or partnered	1589 (78.1)
	Single/separated/widowed	447 (22.0)
Children	None	611 (30.0)
	One or more	1424 (70.0)
Education	High school or below	498 (24.3)
	Vocational training	338 (16.5)
	University	1218 (59.3)
Employment	Part or full time employed	1127 (54.8)
	Retired due to age or disability	521 (25.3)
	Other	409 (19.9)
Type of MS	Relapsing remitting	1290 (62.8)
	Primary progressive	140 (6.8)
	Secondary progressive	225 (11.0)
	Other/unknown	398 (19.4)
Years since diagnosis	(Median and interquartile range)	6 (3–12)
Level of disability	None-mild	1151 (56.2)
	Moderate	698 (34.1)
	Severe	200 (9.8)
Clinical fatigue	Yes	1255 (65.7)
	No	656 (34.3)
Positive screen for depression	Yes	377 (19.0)
	No	1607 (81.0)
Taking antidepressants	Yes	456 (22.5)
	No	1571 (77.5)
Currently using disease modifying drug	Yes	1039 (51.3)
	No	988 (48.7)

^a^N varies due to non-completion

A comparison of those completing sexual function items (to yield a total score) and those not completing this part of the MSQOL-54 revealed participants were more likely to complete sexual function items if they were younger (mean age 45.0 vs 48.0, *p* < .001), more recently diagnosed (mean years since diagnosis 8.5 vs 9.4, *p* = .028), male (completed: male, 95.3 % vs female, 87.7 %, *p* < .001), and partnered (completion: 88.9 %, vs single, 68.8 %; separated/divorced/widowed, 70.4 %; *p* < .001).

Overall, 54.5 % (1123, 95 % CI 52.3–56.6 %) were categorised as having SD (49.7 % of men and 55.6 % of women, *p* = .036). Lack of sexual interest was somewhat or very much a problem for 39.6 % (29.8 % of men and 41.8 % of women, *p* < .001); difficulty with erection or lubrication was somewhat or very much a problem for 32.8 % (40.7 % of men and 30.9 % of women); 34.9 % reported that having an orgasm was somewhat or very much a problem (30.1 % of men and 36.0 % of women, *p* = .029); and 21.5 % said that satisfying a sexual partner was somewhat or very much a problem (28.9 % of men and 19.7 % of women, *p* < .001). For the single item on overall satisfaction, close to half the sample (*n* = 889, 43.7 %) were satisfied or very satisfied with their sexual function in the previous 4 weeks, with no significant gender difference. Those with SD were less likely to be satisfied compared to those without SD (23.9 % vs 76.1 %, *p* < .001). The median total sexual function score for the entire sample was 75.0 (IQR 41.7–91.7); and the mean was 66.3 ([95 % CI 65.0–67.7]).

### Sexual dysfunction

Variables that showed significant unadjusted associations with SD were age, gender, marital status, children, employment, education, disability, depression, antidepressant use, fatigue, smoking status, level of alcohol use, level of physical activity, omega 3 supplementation, frequency of meditation, BMI and diet score. The final regression model (Table 2) showed that screening positive for depression, clinical fatigue, being older, being retired due to age or disability and using an antidepressant were each factors associated with higher odds for SD. Not having a partner and having a healthier diet were associated with lower odds for SD.

**Table 2:** Factors associated with sexual dysfunction

Variable	Category	Adj OR	*P*	95 % CI	
Age		1.03	<0.001	1.01	1.04
Gender	Female	Ref			
	Male	0.77	0.063	0.59	1.01
Marital status	Married/cohabiting/ partnered	Ref			
	Single	0.51	<0.001	0.35	0.75
	Separated/divorced/widower	0.37	<0.001	0.26	0.53
Number of children	0	Ref			
	1	1.40	.058	.99	1.98
	2	1.19	.262	.88	1.59
	3 or more	0.72	.061	0.52	1.02
Employment	Employed full or part time	Ref			
	Student or stay at home carer	1.17	0.380	0.83	1.65
	Unemployed	0.95	0.816	0.63	1.44
	Retired due to age or disability	1.54	0.005	1.14	2.10
Level of disability	No or some disability	Ref			
	Gait/cane disability	1.29	0.053	1.00	1.67
	Major mobility support	1.00	0.988	0.65	1.55
Clinically significant fatigue	No	Ref			
	Yes	1.61	<0.001	1.26	2.05
Depression screen	Negative	Ref			
	Positive	1.92	<0.001	1.42	2.60
Smoking status	Never smoked	Ref			
	Previously smoked	1.08	0.506	0.86	1.35
	Current smoker	1.38	0.083	0.96	1.98
Physical activity	Low active	Ref			
	Moderate active	0.93	0.557	0.71	1.20
	High active	0.91	0.509	0.69	1.20
Body mass index	Normal	Ref			
	Underweight	0.64	0.102	0.37	1.09
	Overweight	0.93	0.601	0.72	1.21
	Obese	1.26	0.125	0.94	1.70
Dietary score (0–100)		0.99	0.023	0.98	1.00
Prescription anti-depressant use	No	Ref			
	Yes	1.43	0.010	1.09	1.89

*N* = 1720, LR CHI2(23) = 262.6, *p* < .001, Pseudo R-squared = .111

### Satisfaction with sexual function

Variables that showed significant unadjusted associations with satisfaction with sexual function were age, gender, employment, disability, depression, use of antidepressants, fatigue, smoking status, level of alcohol use, level of physical activity, omega 3 supplementation, BMI and diet score. The final regression model (Table 3) showed that increasing age, screening positive for depression, clinical fatigue, and using antidepressants were associated with lower odds, while having a high level of physical activity was associated with higher odds of being satisfied with sexual function.

**Table 3:** Factors associated with satisfaction with sexual function

		Adj OR	*P*	95 % CI	
Age		0.98	<0.001	0.96	0.99
Gender	Male	Ref			
	Female	1.12	0.401	0.86	1.45
Employment	Employed full or part time	Ref			
	Student or stay at home carer	0.90	0.509	0.64	1.24
	Unemployed	1.14	0.519	0.77	1.69
	Retired due to age or disability	0.85	0.262	0.63	1.13
Level of disability	No or some disability	Ref			
	Gait/cane disability	0.82	0.106	0.64	1.05
	Major mobility support	0.92	0.711	0.61	1.40
Clinically significant fatigue	No	Ref			
	Yes	0.72	0.006	0.58	0.91
Depression screen	Negative	Ref			
	Positive	0.49	<0.001	0.37	0.66
Physical activity	Low active	Ref			
	Moderate active	1.24	0.093	0.97	1.59
	High active	1.61	<0.001	1.23	2.09
Prescription anti-depressant use	Yes	0.72	0.014	0.56	0.94
	No	Ref			

*N* = 1754. LR CHI2(12) = 163.95, *p* < .001. PseudoR-squared = .0683

### Sexual function scale

Variables that showed significant unadjusted associations with sexual function score were age, gender, marital status, number of children, employment, level of education, disability, depression, antidepressant use, fatigue, smoking status, level of alcohol use, level of physical activity, omega 3 supplementation, BMI and diet score The final regression model (Table 4) showed that increasing age, being moderately or severely disabled, being retired due to age or disability, screening positive for depression, clinically significant fatigue and antidepressant use were independently associated with a lower score on the sexual function scale, while not having a partner, and having a high level of physical activity was associated with a higher score.

**Table 4:** Factors associated with sexual function scale score

		Adj Coef.	*P*	95 % CI	
Age		−0.47	<0.001	−0.62	−0.31
Gender	Male	Ref			
	Female	2.06	0.24	−1.361	5.48
Marital status	Married/cohabiting/ partnered	Ref			
	Single	7.95	<0.001	3.64	12.25
	Separated/divorced/ widower	12.56	<0.001	7.99	17.14
Employment status	Employed full or part time	Ref			
	Student or stay at home carer	−1.51	0.501	−5.92	2.89
	Unemployed	1.27	0.637	−4.02	6.57
	Retired due to age or disability	−5.19	0.007	−8.98	−1.40
Level of disability	No or some disability	Ref			
	Gait/cane disability	−6.25	<0.001	−9.55	−2.94
	Major mobility support	−5.91	0.032	−11.31	−0.51
Clinically significant fatigue	No	Ref			
	Yes	−5.41	0.001	−8.57	−2.26
Depression screen	Negative	Ref			
	Positive	−13.77	<0.001	−17.41	−10.14
Prescription anti-depressant use	Yes	−9.40	<0.001	−12.74	−6.06
	No	Ref			
Physical activity	Low active	Ref			
	Moderate active	3.11	0.07	−0.19	6.41
	High active	4.11	0.024	0.54	7.67

*N* = 1756, F(14, 1741) = 31.01, *p* < =0.001, Adj R-squared = 0.1932

### Subgroup analyses

Further analyses were performed with the above regression analyses repeated for only the subgroup with a partner. As results did not differ meaningfully from the full case analysis, we do not report these findings here.

## Discussion

Sexual function is an important issue for PwMS. Young adults with MS particularly may see SD as the most negative feature of the illness, given the variety of disturbances of sexual function, and the indirect effects on mental health, quality of life and intimate relationships, at a time of life when sexual activity may be at a peak and seen as particularly important. Sexual function is however often not a standard part of the consultation with healthcare professionals for PwMS [27], despite calls for its inclusion [28–30], and is therefore frequently underdiagnosed [31].

Our data on a large sample of PwMS world-wide confirmed that SD is common, with over half of the sample reporting one or more problems with sexual function, and that SD is associated with lower satisfaction with sexual function. Lack of sexual interest was the most common problem in women while difficulty with erection was the most common in men. The prevalence of SD in our study was lower than in previous reports. One study found that 73.1 % of PwMS reported one or more sexual disturbances. This was nearly double compared to those with other chronic diseases (39.2 %) and many times higher than healthy controls (12.8 %) [7]. Others suggest a higher prevalence in the general population, at about 40–45 % of women and 20–30 % of men [11]. However, definitions are not directly comparable between studies, and our sample is not likely to be representative of the general MS population. It is probable that our sample would have lower levels of SD than other studies given that our sample was notably young, educated and had a lower level of disability [25].

For the sexual function scale of the MSQOL-54 score, we showed significant independent associations with age, marital status, employment status, level of disability, physical activity, clinical fatigue, screening positive for depression and antidepressant use. Age, depression, antidepressant use, fatigue, and level of physical activity were also independent predictors associated with satisfaction with sexual function. Similarly, clinical fatigue, depression and antidepressant use were independent determinants of the presence of SD, along with age, marital, and employment status, as well as the modifiable factor, diet.

This suggests that sexual function and dysfunction for PwMS have very complex determinants related to an interplay of individual, lifestyle and disease characteristics, strongly influenced by the presence of depression, the use of antidepressants, and fatigue. We have previously demonstrated modifiable lifestyle factors are important predictors in their own right of fatigue [32] and depression risk [33], and may therefore indirectly affect sexual function and satisfaction with sexual function. Here, we were able to demonstrate that diet and level of physical activity are associated with these outcomes even after adjusting for other relevant factors.

Although smoking showed significant unadjusted associations with our outcomes, in the final regression models, it did not reach a significance level of <.05. Smoking has previously been linked with erectile dysfunction [34], probably mediating its effects via microvascular circulatory impairment, although the mechanism in women has not been well studied. Regular exercise would likely counteract this mechanism, and our data suggest an association with sexual function score, and satisfaction with sexual function after adjusting for depression and fatigue. Similarly, better diet may have beneficial effects directly on the microvasculature, but also improve satisfaction with sexual function through indirect effects such as amelioration of metabolic syndrome, known to have adverse effects on sexual function [35, 36].

To our knowledge, only two studies have thus far discussed the effect of lifestyle modification on SD [11, 37]. These studies suggest that increasing physical activity improves symptoms of SD, however it is possible that positive effects of exercise on mood mediated the observed improvements. Our data suggest these effects are independent determinants of SD. Modifying other factors such as smoking, obesity and alcohol consumption may also improve SD if managed early in life, before middle age [37].

SD is very common in people with depression in the general population, who have a 50 to 70 % risk [24]; this relationship appears to be bi-directional, in that people with SD have a greatly heightened risk of depression [24]. As depression occurs very commonly in PwMS [38, 39], with a lifetime prevalence of around 50 % [40], this contributes to the very high prevalence of SD in PwMS. A number of studies have shown that quality of life with respect to SD is lower in PwMS who have depression [21, 39]. PwMS should be screened for depression as early prevention and treatment of depression has many benefits including a potential benefit for people who experience SD [39]. However, antidepressant use was clearly associated with the presence of sexual dysfunction, lower odds for satisfaction with sexual function and lower sexual function score, independent of the other factors including screening positive for depression.

Fatigue also has a complex relationship with depression, and so may be expected to be associated with SD for PwMS. Our study however confirmed associations with the outcomes that were independent of and additional to the associations with depression. Fatigue has been shown to be significantly associated with SD in a number of studies [16, 19, 23, 29, 41]. A significant relationship was found between SD and the presence of physical disorders impeding sexual activity, in particular fatigue [20], and although reported by people with a range of chronic disease, appears to be a particular problem for PwMS [7].

Associations between SD and various MS-related clinical and socio-demographic variables have been investigated in other studies [19, 20, 23, 42]. Meaningful correlations were found between SD and age, male gender, low education level, unemployment, length of disease, length of marriage, anxiety, age of husband, physical ability, cognitive deterioration, length of medication use, primary progressive MS, frequency of intercourse, number of pregnancies and number of children [16, 19, 20]. While age, marital status, level of education, employment status, number of children, and level of disability were associated with our sexual health outcomes, we did not find an independent association of gender or medication use in our data.

There is potential for a lifestyle risk factor modification approach to the management of SD in PwMS, given the strong associations of better lifestyle behaviours with reduced depression risk [33] and fatigue [32], both known to adversely affect sexual function [22]. In type 2 diabetes, another chronic disease with strong lifestyle associations, intensive lifestyle intervention in obese women resulted in a significantly greater proportion remaining sexually active, improvements in sexual function, and greater likelihood of remission of SD at 1 year [43]. Lifestyle approaches to management of SD in PwMS are however uncommon, and there is little supporting research.

Given the important role of depression in SD, our data suggest that a more holistic approach to the management of depression in PwMS is required. This is particularly important given the associations we have demonstrated of antidepressant use with SD, sexual satisfaction and overall sexual function. Others have previously called for judicious use of antidepressant medication on a background of routine use of lifestyle modification for the treatment of depression [44]. A drug-only approach to management of depression in PwMS appears a poor therapeutic approach. Attention to lifestyle risk factors, psychosocial interventions and psychological therapy may decrease depression risk in their own right but also increase sexual satisfaction [45]. From a practical viewpoint, PwMS often have worse fatigue and particularly low energy in the afternoon and night time, increasing the likelihood of SD and avoidance of sexual activities. On this basis, PwMS may also be advised to engage in sexual activity earlier in the day to reduce SD due to fatigue [41].

### Limitations

All data in our study were self-reported, and therefore were unable to be verified, although previously validated tools were used where possible. We did not measure the presence of depression directly, but screened for depression risk. SD was determined by categorising participants in two groups depending on their responses to items on the sexual function scale of the MSQOL-54; this method has not previously been used or validated, but does allow for future studies to directly compare data with ours. Participants were English speaking and able to complete the survey online, and most were young women. This sample may therefore not be generalizable to all PwMS, despite the variety of backgrounds of participants and size of the sample. Finally, our study examined associations and is cross-sectional, hence we cannot assert causality.

## Conclusions

Our data confirm that SD is common in the MS population, associated with decreased satisfaction with sexual function. Sexual function and dysfunction for PwMS have very complex determinants, related to an interplay of individual, lifestyle and disease characteristics, strongly influenced by the presence of depression, antidepressant use and fatigue. The modifiable factors physical activity and diet were associated with sexual function and satisfaction after adjusting for relevant other variables, raising potential for secondary prevention. Further research should confirm the nature of the associations of modifiable factors identified in this study, as well as the drivers of fatigue and depression for PwMS, as this may assist in improving SD.

## Abbreviations

BMI: Body mass index
DHQ: Dietary Habits Questionnaire
DMDs: Disease-modifying drugs
EDSS: Expanded Disability Status Scale
HOLISM: Health Outcomes and Lifestyle In a Sample of people with Multiple sclerosis
HRQOL: Health-related Quality of Life
IPAQ: International Physical Activity Questionnaire
MS: Multiple sclerosis
MSQOL-54: Multiple Sclerosis Quality Of Life-54 instrument
NARCOMS: North American Research Committee on Multiple Sclerosis
PDDS: Patient Determined Disease Steps
PwMS: People with multiple sclerosis
SD: Sexual dysfunction
WHO: World Health Organization

## References

[1] Kingwell E, Marriott JJ, Jetté N (2013). Incidence and prevalence of multiple sclerosis in Europe: a systematic review. BMC Neurol.

[2] Foley FW, Zemon V, Campagnolo D (2013). The Multiple Sclerosis Intimacy and Sexuality Questionnaire -- re-validation and development of a 15-item version with a large US sample. Mult Scler.

[3] Schairer LC, Foley FW, Zemon V (2014). The impact of sexual dysfunction on health-related quality of life in people with multiple sclerosis. Mult Scler.

[4] Dupont S (1995). Multiple sclerosis and sexual functioning - a review. Clin Rehabil.

[5] Calabro RS, De Luca R, Conti-Nibali V, Reitano S, Leo A, Bramanti P (2014). Sexual dysfunction in male patients with multiple sclerosis: a need for counseling!. Int J Neurosci.

[6] Celik DB, Poyraz EC, Bingol A, Idiman E, Ozakbas S, Kaya D (2013). Sexual dysfunction in multiple sclerosis: gender differences. J Neurol Sci.

[7] Zorzon M, Zivadinov R, Bosco A (1999). Sexual dysfunction in multiple sclerosis: a case-control study. I. Frequency and comparison of groups. Mult Scler.

[8] Foley FW, LaRocca NG, Sanders AS, Zemon V (2001). Rehabilitation of intimacy and sexual dysfunction in couples with multiple sclerosis. Mult Scler.

[9] Kessler TM, Fowler CJ, Panicker JN (2009). Sexual dysfunction in multiple sclerosis. Expert Rev Neurother.

[10] Basson R, Leiblum S, Brotto L (2003). Definitions of women’s sexual dysfunction reconsidered: advocating expansion and revision. J Psychosom Obstet Gynaecol.

[11] Lewis RW, Fugl-Meyer KS, Bosch R (2004). Epidemiology/risk factors of sexual dysfunction. J Sex Med.

[12] Lewis RW, Fugl-Meyer KS, Corona G (2010). Definitions/epidemiology/risk factors for sexual dysfunction. J Sex Med.

[13] World Health Organisation. International Statistical Classification of Diseases and Related Health Problems 10th Revision (ICD-10)-WHO Version for 2016. 2015; http://apps.who.int/classifications/icd10/browse/2016/en#!/F52.0. Accessed 12 Oct 2015.

[14] Stenager E, Stenager EN, Jensen K (1992). Sexual aspects of multiple sclerosis. Semin Neurol.

[15] Vickrey BG, Hays RD, Harooni R, Myers LW, Ellison GW (1995). A health-related quality of life measure for multiple sclerosis. Qual Life Res.

[16] Merghati-Khoei E, Qaderi K, Amini L, Korte JE (2013). Sexual problems among women with multiple sclerosis. J Neurol Sci.

[17] Vas CJ (1969). Sexual impotence and some autonomic disturbances in men with multiple sclerosis. Acta Neurol Scand.

[18] Valleroy ML, Kraft GH (1984). Sexual dysfunction in multiple sclerosis. Arch Phys Med Rehabil.

[19] Tepavcevic DK, Kostic J, Basuroski ID, Stojsavljevic N, Pekmezovic T, Drulovic J (2008). The impact of sexual dysfunction on the quality of life measured by MSQoL-54 in patients with multiple sclerosis. Mult Scler.

[20] Zivadinov R, Zorzon M, Bosco A (1999). Sexual dysfunction in multiple sclerosis: II. Correlation analysis. Mult Scler.

[21] Janardhan V, Bakshi R (2002). Quality of life in patients with multiple sclerosis: the impact of fatigue and depression. J Neurol Sci.

[22] Darija KT, Tatjana P, Goran T (2015). Sexual dysfunction in multiple sclerosis: A 6-year follow-up study. J Neurol Sci.

[23] Demirkiran M, Sarica Y, Uguz S, Yerdelen D, Aslan K (2006). Multiple sclerosis patients with and without sexual dysfunction: are there any differences?. Mult Scler.

[24] Atlantis E, Sullivan T (2012). Bidirectional association between depression and sexual dysfunction: a systematic review and meta-analysis. J Sex Med.

[25] Hadgkiss EJ, Jelinek GA, Weiland TJ, Pereira NG, Marck CH, van der Meer DM (2013). Methodology of an International Study of People with Multiple Sclerosis Recruited through Web 2.0 Platforms: Demographics, Lifestyle, and Disease Characteristics. Neurol Res Int.

[26] Lindsey C, Sheather SJ (2010). Variable selection in linear regression. Stata J.

[27] Calabro RS, Russo M (2015). Sexual Dysfunction and Depression in Individuals with Multiple Sclerosis: Is there a Link?. Innov Clin Neurosci.

[28] Hawker KS, Frohman EM (2001). Bladder, Bowel, and Sexual Dysfunction in Multiple Sclerosis. Curr Treat Options Neurol.

[29] Nortvedt MW, Riise T, Myhr KM, Landtblom AM, Bakke A, Nyland HI (2001). Reduced quality of life among multiple sclerosis patients with sexual disturbance and bladder dysfunction. Mult Scler.

[30] Orasanu B, Frasure H, Wyman A, Mahajan ST (2013). Sexual dysfunction in patients with multiple sclerosis. Mult Scler Relat Disord.

[31] Lew-Starowicz M, Gianotten WL (2015). Sexual dysfunction in patients with multiple sclerosis. Handb Clin Neurol.

[32] Weiland TJ, Jelinek GA, Marck CH (2015). Clinically significant fatigue: prevalence and associated factors in an international sample of adults with multiple sclerosis recruited via the internet. PLoS One.

[33] Taylor KL, Hadgkiss EJ, Jelinek GA (2014). Lifestyle factors, demographics and medications associated with depression risk in an international sample of people with multiple sclerosis. BMC Psychiatry.

[34] Kovac JR, Labbate C, Ramasamy R, Tang D, Lipshultz LI (2015). Effects of cigarette smoking on erectile dysfunction. Andrologia.

[35] Saenz Medina J, Carballido Rodriguez J (2015). Review of the pathophysiological aspects involved in urological disease associated with metabolic syndrome. Actas Urol Esp.

[36] Moyad MA, Park K (2012). What do most erectile dysfunction guidelines have in common? No evidence-based discussion or recommendation of heart-healthy lifestyle changes and/or Panax ginseng. Asian J Androl.

[37] Derby CA, Mohr BA, Goldstein I, Feldman HA, Johannes CB, McKinlay JB (2000). Modifiable risk factors and erectile dysfunction: can lifestyle changes modify risk?. Urology.

[38] Lew-Starowicz M, Rola R (2014). Correlates of sexual function in male and female patients with multiple sclerosis. J Sex Med.

[39] Wang JL, Reimer MA, Metz LM, Patten SB (2000). Major depression and quality of life in individuals with multiple sclerosis. Int J Psychiatry Med.

[40] Siegert RJ, Abernethy DA (2005). Depression in multiple sclerosis: a review. J Neurol Neurosurg Psychiatry.

[41] Zivadinov R, Zorzon M, Locatelli L (2003). Sexual dysfunction in multiple sclerosis: a MRI, neurophysiological and urodynamic study. J Neurol Sci.

[42] Mattson D, Petrie M, Srivastava DK, McDermott M (1995). Multiple sclerosis. Sexual dysfunction and its response to medications. Arch Neurol.

[43] Wing RR, Bond DS, Gendrano IN (2013). Effect of intensive lifestyle intervention on sexual dysfunction in women with type 2 diabetes: results from an ancillary Look AHEAD study. Diabetes Care.

[44] Sarris J, O’Neil A, Coulson CE, Schweitzer I, Berk M (2014). Lifestyle medicine for depression. BMC Psychiatry.

[45] Cordeau D, Courtois F (2014). Sexual disorders in women with MS: assessment and management. Ann Phys Rehabil Med.

